# Minimum and maximum energy for crystals of magnetic dipoles

**DOI:** 10.1038/s41598-020-76029-x

**Published:** 2020-11-05

**Authors:** Josep Batle, Orion Ciftja

**Affiliations:** 1 Departament de Física, Universitat de les Illes Balears, 07122 Palma de Mallorca, Balearic Islands Spain; 2 Department of Physics, Prairie View A&M University, Prairie View, TX 77446 USA

**Keywords:** Materials science, Physics

## Abstract

Properties of many magnetic materials consisting of dipoles depend crucially on the nature of the dipole–dipole interaction. In the present work, we study systems of magnetic dipoles where the dipoles are arranged on various types of one-dimensional, two-dimensional and three-dimensional lattices. It is assumed that we are in the regime of strong dipole moments where a classical treatment is possible. We combine a new classical numerical approach in conjuncture with an ansatz for an energy decomposition method to study the energy stability of various magnetic configurations at zero temperature for systems of dipoles ranging from small to an infinite number of particles. A careful analysis of the data in the bulk limit allows us to identify very accurate minimum and maximum energy bounds as well as ground state configurations corresponding to various types of lattices. The results suggest stabilization of a particularly interesting ground state configuration consisting of three embedded spirals for the case of a two-dimensional hexagonal lattice.

## Introduction

Significant progress in experimental techniques in the past decade has made possible the realization of dipolar gases consisting of molecules that have large dipole moments^1–3^. For such systems, formation of a classical crystal of dipolar particles is expected at those temperatures where the thermal energy is weak relative to the dominant characteristic dipolar energy. The behavior of the system under these conditions is then solely dominated by dipolar interactions. The focus of the present work is to study the properties of dipolar systems exactly in this regime. This means that we ignore thermal effects and treat the system as being at absolute zero temperature. The geometry considered is one where the dipolar particles are localized in one-dimensional (1D), two-dimensional (2D) and three-dimensional (3D) lattices.

Differently from the famous Wigner crystal state of electrons that has been predicted for systems of particles with a Coulomb interaction at low density^4,5^, the crystal phases of particles with dipolar interaction should be found at high density. Earlier work on this topic has considered systems of dipoles oriented perpendicular to the motional plane and shed light on their properties by treating them both classically^6–10^ and quantum mechanically^11–16^. This approach leads to a dipole–dipole interaction that depends only on the relative separation distance between them and neglects the angular degrees of freedom. By avoiding to consider the orientational dependence, these treatments represent a strong oversimplification of the problem.

Another line of investigation has been the study of dipole systems localized on fixed lattice sites, for instance 2D lattices^17–20^. These works use a variety of different orientations of the dipoles with respect to the plane of the lattice. In this case the dipole–dipole interaction is no longer cylindrically symmetric on the plane and, as a result, can be highly anisotropic. This arrangement may lead to interesting striped systems as indicated in several studies^21–23^. Depending on a variety of other factors, dipolar interactions may also lead to new unexpected properties like the roton minimum in helium^24^. Similar effects have also been observed and studied in externally oriented magnetic colloids^25–27^.

Overall, there is a huge scientific interest on studies of the properties of 2D magnetic systems with continuous spin degrees of freedom. At a finite temperature, such systems exhibit a rich spectrum of thermal behavior because of the strong competition between thermal fluctuations and correlation effects. More complex behavior emerges when an additional anisotropic dipolar interaction is present. It is expected that, for such circumstances, a discrete symmetry emerges. When such discrete symmetry is spontaneously broken the result is that the system is led to a low temperature ordered phase. However, observation of such phases and, in general, experimental realization of 2D dipolar systems in crystalline materials is difficult since the dipolar coupling is usually much weaker than the exchange interaction. The apparent reason is that in most physical systems the dipole interaction is a small perturbation against the background of the stronger interaction. Thus, in magnetically ordered crystals, the ground state of the spin system is determined by exchange interaction, and the role of the dipole–dipole interaction reduces to stimulation of the formation of the domain structure, and to other effects that are extremely important but do not influence the character of the magnetic ordering.

Both classical and quantum studies of interacting dipolar systems of particles are very challenging because of the interplay of so many factors and various outcomes. However, in those regimes where a classical treatment is possible, the study of classical properties of crystals is a first crucial step to get a better understanding of the more complex properties of the quantum cases^28,29^. In the current work, we assume that the dipole moments are large, therefore, a classical treatment is suitable and accurate.

One of the first attempts to formulate a theory of dipole interaction in crystals was carried out by Luttinger and Tisza^30^. Their classic paper developed the principles of investigation of the ground state of such systems (as well as the maximum and intermediate configuration energies). Their elegant method led to the solution of an eigenvalue–eigenstate diagonalization problem for properly chosen finite multi-vector spaces. This very same method was later extended to systems with quadrupole interaction by Nagai and Nakamura^31^. These two pioneering works succeeded on explaining various experimental results for dipolar compounds that were amenable to a classical treatment.

As explained earlier, there is a renewed interest to better understand the properties of dipole systems, revamping a lot of attention on classical treatments. In the present contribution we adopt a new perspective to study systems of dipolar particles placed at the sites of a 1D lattice, a 2D simple square, hexagonal and honeycomb lattice, as well as a 3D simple cubic lattice. The novelty of our work extends in three directions: (i) A new computational approach that leads to very accurate energy values in the bulk limit; (ii) New results that apply to rather challenging systems of dipoles in 2D hexagonal and honeycomb lattices; and (iii) A new energy decomposition formula that is very accurate in the bulk limit. For the sake of completeness, we also show known results for a 1D lattice, a 2D simple square lattice and 3D simple cubic lattice with the sole purpose of gauging the accuracy of the presently employed computational method. The current method that relies on a systematic numerical computation of the minimum and maximum energies by *growing finite samples with an increasing size that preserves the scale* allows us not only to recover Luttinger and Tisza’s previous results in 3D, but also to obtain new ones in 2D such as the ground state of a rather complex planar hexagonal lattice. Furthermore, a novel finding of our calculations is that the total bulk energy value of the system can be heuristically shown that it can be systematically divided into contributions that account for the volume and surface of the system (much like the liquid drop model in nuclear physics) as the total number of particles tends to infinity. It is also worthwhile pointing out that within the framework of our method there is no need to resort to solutions of eigenvalue–eigenstate problems, which means that this method is computationally less expensive than other methods such as Monte Carlo, etc. Finally, it is also important to note the remarkable accuracy of an energy decomposition formula that we uncovered in this work allowing us to obtain the minimum energy in the bulk limit. The present work is divided as follows. In Model section we describe the model and show how (minimum and maximum) energies are computed for a 2D scenario (the formalism can be easily extended to any other dimension). In One-dimensional case section we present the results for the trivial 1D case. The 2D case (for different planar lattices) including complex hexagonal and honeycomb lattices is studied in detail in Two-dimensional case section. Three-dimensional case section lists some results for the 3D case scenario (for a simple cubic lattice). Finally, some conclusions are drawn in Conslusions section to reiterate the novelty and importance of this work from the perspective of technological applications in fields like spintronics or topological matter.

## Model

We consider a model where all (magnetic) point dipoles are assumed to be identical and have a given dipole moment with magnitude $\mu$. For simplicity, we discuss here in detail the model for the case of a 2D crystal lattice. The formalism and all considerations can be straightforwardly extended to other dimensions. For the case of a 2D lattice, it is assumed that the dipoles lie on the 2D plane of the lattice with no extra degree of freedom perpendicular to it. This arrangement is similar to that of a classical XY spin system^32^ in a 2D lattice. The dipoles have all initially different random orientations while fixed at the sites of the 2D lattice. The direction of each dipole moment is given in terms of a polar angle, $\theta$. For the case of XY dipoles in a 2D lattice, the dipole moment itself, $\vec {m}$ is described by a vector with two components of the form:

1 $$\begin{aligned} \vec {m} = \mu \begin{pmatrix} \cos (\theta ) \\ \sin (\theta ) \end{pmatrix}. \end{aligned}$$

Obviously, in 3D, the dipoles have all their degrees of freedom and their dipole moment orientations are given in terms of two angles. The expression for the potential energy of interaction between two point dipoles is readily available in the literature. For instance, the formula for this form of energy is given in Pg. 378 of Zangwill’s book^33^ including a nice discussion of all the preceding steps that lead to such a result.

**Figure 1:**
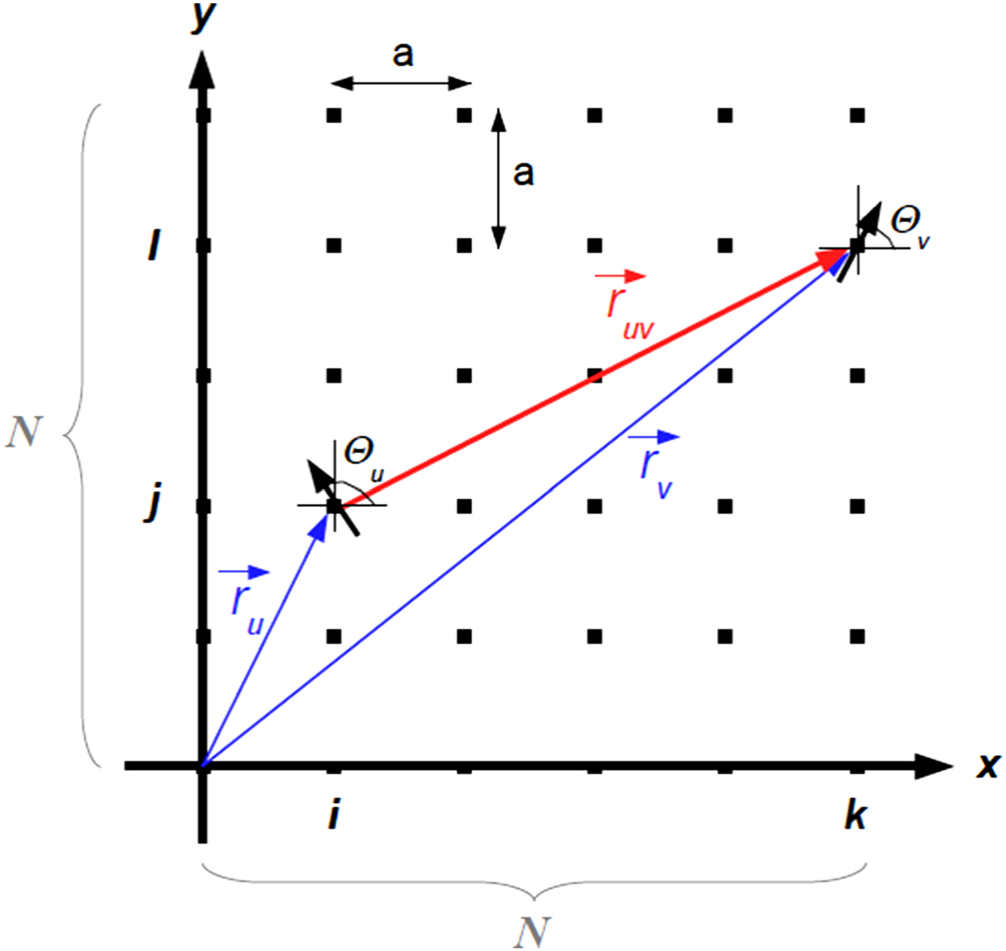
Schematic presentation of the distribution of magnetic dipoles in a 2D square lattice. See text for details. (Image created with OpenOffice 4.1.5; https://www.openoffice.org).

In our case, the dipole–dipole interaction potential energy between any two point dipoles, $\vec {m}_u$ and $\vec {m}_v$ localized, respectively, at two different sites $\vec {r}_u$ and $\vec {r}_{v}$ $( \vec {r}_u \ne \vec {r}_{v})$ of a simple 2D square lattice can be written as:

2 $$\begin{aligned} E_{u,v} = \frac{\mu _0}{4\pi } \bigg [ \frac{\vec {m}_u\cdot \vec {m}_v}{r_{uv}^3}- \frac{3 \left( \vec {m}_u\cdot \vec {r}_{uv}\right) \left( \vec {m}_v\cdot \vec {r}_{uv}\right) }{r_{uv}^5} \bigg ], \end{aligned}$$

where $\vec {r}_{uv}=\vec {r}_v -\vec {r}_{u}$ is the separation vector between the two dipoles $\vec {m}_u$ at $\vec {r}_{u}$ and $\vec {m}_v$ at $\vec {r}_v$, the quantity $r_{uv}=|\vec {r}_{uv}|$ is the magnitude of their separation distance and $\mu _0$ is the magnetic permeability of the free space. For a simple 2D square lattice, the unit cell is a square of size $a\times a$. The expression for the energy in Eq. (2) contains two contributions which we call, from now on, the *first term* and the *second term* in the interaction energy expression, the latter one including a minus sign. The vector $\vec {r}_{u}$ is equal to $(a\,i,a\,j)$, and similarly, $\vec {r}_{v}$ is equal to $(a\,k,a\,l)$ where *i*, *j*, *k*, *l* are integers. Values of index *u* are uniquely determined by the pair of integers (*i*, *j*), whereas the value of *v* is given by (*k*, *l*). A schematic setup of a system of $N\times N$ dipoles in a regular 2D square lattice is shown in Fig. 1.

The total energy of a system containing $N \times N$ dipoles is calculated as:

3 $$\begin{aligned} E_{N\times N}= \sum _{\{ (u,v) \}} E_{u,v}, \end{aligned}$$

where $\{ (u,v) \}$ (symbolically) represents the sum of all interacting pairs of the dipoles in the system. Since final energy expressions are more conveniently expressed in dimensionless units, we introduce the quantity $U_{N\times N}$ which represents the total dimensionless energy, defined as:

4 $$\begin{aligned} U_{N\times N} \equiv {E_{N\times N}}/\left( {\frac{\mu _0}{4\pi } \frac{\mu ^2}{a^3}}\right) . \end{aligned}$$

As a result, the total (dimensionless) energy of the system with $N\times N$ magnetic dipoles can be written as:

5 $$\begin{aligned} U_{N\times N}=&\sum _{i<j}^{N-1} \,\sum _{k<l}^{N-1} \frac{1}{[(i-k)^2+(j-l)^2]^{3/2}} \nonumber \\&\times \bigg [\cos (\theta _{ij}-\theta _{kl})\, \nonumber \\&-\,3\bigg ( \cos \theta _{ij} \frac{i-k}{\sqrt{(i-k)^2+(j-l)^2]}}\,\nonumber \\&+\,\sin \theta _{ij} \frac{j-l}{\sqrt{(i-k)^2+(j-l)^2}} \bigg ) \nonumber \\&\times \bigg (\cos \theta _{kl} \frac{i-k}{\sqrt{(i-k)^2+(j-l)^2]}}\,\nonumber \\&+\,\sin \theta _{kl} \frac{j-l}{\sqrt{(i-k)^2+(j-l)^2}} \bigg )\bigg ], \end{aligned}$$

where $\theta _{ij}$ and $\theta _{kl}$, namely, $\theta _{u}$ and $\theta _{v}$ stand for the angles of dipoles at positions $\vec {r}_u$ and $\vec {r}_v$, respectively (See Fig. 1). The problem consists in finding the absolute minimum (and maximum) of the total energy in Eq. (5) for systems with $N\times N\,=\,N^2$ independent variables. Strictly speaking, and due to the rotational invariance of the system, the minimization should take place over a total of $N^2-1$ variables or angles.

The classical extremal energy states of a magnetic dipole system is one of equilibrium in which no torque should act on any given dipole. In the present study, *the equilibrium states for systems with a finite number of dipoles* are found numerically by considering an arbitrary large number of dipoles. Of course, the situation becomes intractable in the bulk limit when the number of dipoles tends to infinity. Nevertheless, it is expected that the system of interacting magnetic dipoles has its total energy bounded from below and from above. Thus, one can formally write two energy bounds ($B^{min}$ and $B^{max}$) so that, for any sistem size, one has:

6 $$\begin{aligned} B^{\min }_{N\times N} \,\le U_{N\times N} \,\le B^{\max }_{N\times N}, \end{aligned}$$

where $B^{\min }_{N\times N}$ is the lower bound to $U_{N\times N}$ and, similarly, $B^{\max }_{N\times N}$ is its upper bound. It is our goal to find accurate minimum and maximum energies per dipole that provide precise lower and upper energy bounds as the system tends to thermodynamic limit. The key point that helps finding those magnetic dipole configurations that minimize (maximize) the total energy is the fact that the dipole–dipole interaction has a range shorter ($\propto 1/r^3$) than the Coulomb potential. This observation has immediate consequences, the most important one being that the system of dipoles will tend to resemble specific ensembles of small clusters with only few dipoles. As a result, based on the optimal configurations obtained numerically, we can assume that these clusters constitute only small deviations away from the bulk in a *coarse grained* approach to the bulk limit. This view constitutes the starting basis of our semi-heuristic approach to solving the problem.

There is no formal proof to ascertain the accuracy of this treatment as it could be objected. To such an end, one should provide the energy of the system of dipoles in terms of different contributions accounting not only for individual dipole–dipole interactions, but also between different unit cells. Then, it would remain to prove that this *coarse grained* approach coincides with results from *ab initio* treatments in the thermodynamic limit. However, having said that, we point out that in our work we adopt a more pragmatic view. We rely on the extension of our computational results for finite cases towards the bulk limit by introducing an energy decomposition formula that we hope is a good guess. Then, we point out how well such results extrapolate to the bulk while preserving the maximum symmetry possible, that is, by growing the finite sample isotropically in all coordinates.

## One-dimensional case

The model of dipoles in a 1D regular lattice is the simplest possible and the results are well known. This case even admits an analytic solution in the $N \rightarrow \infty$ limit^34^. Therefore, we report here some energy results only for the sake of completeness without going into much detail for the reasons already given above. In this model it is assumed that a system of *N* dipoles are placed in a 1D regular array with spacing, *a*. It can be easily verified that the minimum possible energy is attained when all dipoles are aligned along the 1D array line. Meanwhile, the maximum possible energy occurs when they alternate their directions. In units of $\mu _0 \,\mu ^2 /(4 \pi a^3)$, the minimum energy per dipole in the thermodynamic limit ($N \rightarrow \infty$) is:

7 $$\begin{aligned} \frac{B_{N}^{min}}{N} = - 2\,\zeta (3) \approx - 2.4041138. \end{aligned}$$

The maximum energy per dipole in such a limit is given by:

8 $$\begin{aligned} \frac{B_{N}^{max}}{N} = \frac{3}{2} \,\zeta (3) \approx 1.8030853, \end{aligned}$$

where $\zeta$(3) is the so called Apéry constant, that is, the Riemann zeta function, $\zeta (z)$ evaluated at $z = 3$. As explained earlier, these results are known in the literature and are not novel. The simplicity of the 1D model is the reason that allows one to easily derive such results in the $N \rightarrow \infty$ limit by starting from the general expression in Eq. (2). What is remarkable in the convergence of the finite size results to the bulk value is that the difference between the mean energy for a fixed number *N* and the asymptotic value diminishes very slowly in the form of $\sim c/N$ with a constant *c* of *O*(1). Clearly, the convergence of the energy results from a finite string to the infinite 1D bulk happens to be slow-going in 1D.

## Two-dimensional case

### Simple square lattice

Let us now consider a system of $N \times N$ dipoles placed on the sites of a simple 2D square lattice. During the minimization of the total energy for the first $N \times N$ plaquettes of interacting dipoles (see Fig. 1), it soon appears that the equilibrium configuration, for a fixed row *j*, is the one corresponding to $\theta = [ (-1)^j -1 ]\frac{\pi }{2}$ for all dipoles in the *i* column. With other words, the $x-y$ plane is divided in alternating stripes of dipoles, each stripe having all its dipoles pointing left or right.

At this juncture, it is important to note that, rather than implementing ab-initio or Monte Carlo-related approaches, the currently employed computational method relies on a systematic numerical computation of the minimum and maximum energies cluster by cluster. This is achieved by growing finite samples of clusters with an increasing size that preserves the scale up to largest possible one that we can reliably identify as the one representing, let’s say, the global energy minimum for that size. Once we reach this stage, we then infer the minimum/maximum equilibrium energy configurations for larger samples by growing them in the most symmetric way possible that is consistent with the pattern identified until we reach a sufficiently large system size where an interpolation of the results in the $N \rightarrow \infty$ limit is accurate. We use this method for all calculations pertaining to considered 2D and 3D lattices having in mind a two-folded objective: (i) Obtain (after fully gauging the accuracy of the method) very accurate values for all concerned energies; and (ii) Show in practice how this growing-sample method works to infer the correct equilibrium configuration when combined with an energy decomposition strategy that we introduced in the current work.

Knowing the equilibrium configuration, becomes now a simple matter of computing the total interaction energy between dipoles as a function of *N*, where it is clear that we grow the (finite) samples of dipoles following a sequence of squares ( with $2\times 2$, $3\times 3$, $\ldots$ dipoles). This dipole configuration results in an energy denoted as $B^{\min }_{N\times N}$, which constitutes, by construction, a lower bound to the real ground state energy value, $U_{N\times N}$. It turns out that the quantity $B^{\min }_{N\times N}$ represents, as we shall see, an excellent lower bound to $U_{N\times N}$, in such a way that we have:

9 $$\begin{aligned} \lim _{N \rightarrow \infty } U_{N\times N}\,/\,N^2\,\longrightarrow \,\lim _{N \rightarrow \infty } B^{\min }_{N\times N}\,/\,N^2. \end{aligned}$$

**Figure 2:**
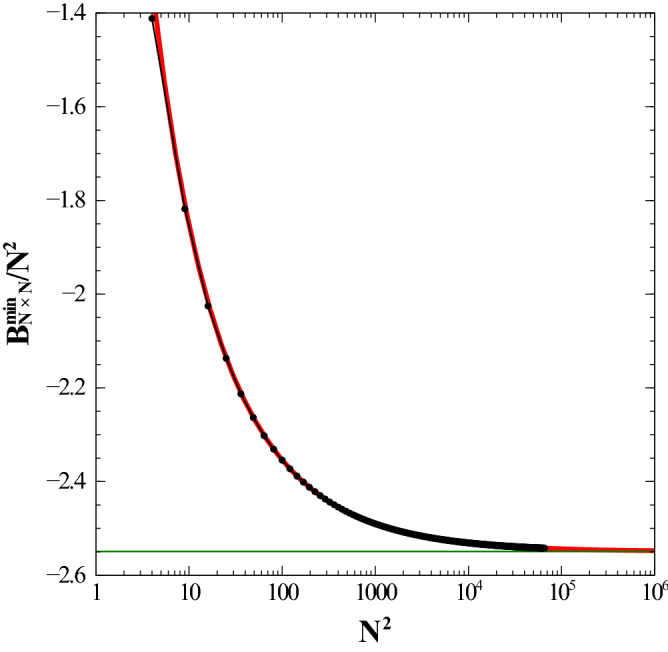
Dependence of the dimensionless energy (per dipole), $B^{\min }_{N \times N}/N^2$ (which represents a lower bound to the true ground state energy) as a function of the total number of dipoles, $N \times N$. The fit is represented by the solid line (red). Logarithmic scale used for the *x* axis. Energy is given in units of ${\frac{\mu _0}{4\pi } \frac{\mu ^2}{a^3}}$. See text for details. (Image created with Veusz 1.18; https://www.veusz.github.io).

The evolution of the minimum energy per dipole, $B^{\min }_{N\times N}/N^2$ as a function of the number $N^2$ of dipoles is shown in Fig.  2. This lower energy bound becomes tighter in the thermodynamic limit where it becomes *exact* in the fashion previously described. We choose to extract information from the data by postulating what we believe are the dominating contributions to the total bulk energy.

To simplify notation, let us denote by “*x*” the total number of dipoles in the plaquette (for example, $x=N^2$ for a 2D square lattice). We then postulate that the total contribution to the minimal energy can be written in the following way:

10 $$\begin{aligned} B^{\min }(x)\,\equiv \,{\mathcal {V}}\,x\,+\, {\mathcal {S}}\,\sqrt{x}\,+\,{\mathcal {R}}(x), \end{aligned}$$

where ${\mathcal {V}}$ stands for the *volumetric* or energy per dipole contribution (the only one we are interested in the $N \rightarrow \infty$ limit), ${\mathcal {S}}$ corresponds to the *surface* energy contribution and ${\mathcal {R}}(x)$ absorbs all other possible finite-size minor contributions. Since ${\mathcal {V}}$ is the quantity we are looking for, all the others should vanish when we calculate $\lim _{x \rightarrow \infty } B^{\min }(x)/x$. The validity of this assumption must be checked by fitting the numerical data. For simplicity, we further assume that ${\mathcal {R}}(x)\,=\,\alpha$ (a constant). The result of the fit for the present case leads to: $\{ {\mathcal {V}}\,=\,-2.54944 \,\pm \,4.386e-008;\, {\mathcal {S}}\,=\,1.83867 \, \pm \,2.21e-005; \, \alpha \,=\,1.16065 \, \pm \, 0.002329\}$.

Note that the minimum energy configuration for a simple 2D square lattice is attained when the angles of the dipoles at the vertices (fixed row *j*, arbitrary column *i*) are $\theta \,=\,[(-1)^j\,-\,1]\pi /2$. Its expression is given as:

11 $$\begin{aligned} U^{min}\,= & {} \, \sum _{i<j}^{N-1} \sum _{k<l}^{N-1} (-1)^{j+l} \, [(i-k)^2\,+\,(j-l)^2]^{-3/2} \nonumber \\&\times \bigg (1\,-\,3\frac{(i-k)^2}{[(i-k)^2\,+\,(j-l)^2]}\bigg ). \end{aligned}$$

The expression above is a rather complex lattice sum but it can be calculated accurately. A direct computation of $U^{min}/N^2$ for large *N* is exactly the previously obtained value $-\;2.54944$ for this desired choice of numerical accuracy. As it can be appreciated, the accuracy of our assumptions is astonishing. The numerical results show that the energy per dipole in the thermodynamic limit, namely the quantity ${\mathcal {V}}$ in Eq. (10), tends to a value $-\;2.54944$, with all decimal digits being *exact*.

We are unaware of previous results noticing the validity of the energy decomposition formula reported in Eq. (10) for the case of dipole–dipole interactions. The fact that the total dipole–dipole energy for a fixed number of dipoles can be decomposed in terms of a several contributions for a large system is very intriguing. A self-energy decomposition procedure can be argued for the case of electrostatic forces, namely, systems that interact with an isotropic Coulomb interaction of $\propto 1/r$ form. However, it would have been a long stretch to echo the same view for the case of a dipolar–dipolar interaction which is highly anisotropic and of $\propto 1/r^3$ form. The argument for the case of a 2D system of point charges in electrostatic equilibrium goes as follows. Assume one has *N* equal point charges, *q* of the same sign confined by a given potential in certain region. These charges will eventually arrange themselves forming an equilibrium pattern. For large systems, the total electrostatic self-energy can be calculated by approximating the density by its continuum limit. The (dimensionless) exact self-energy *U* of a continuous charge distribution given in units of $\propto Q^2/L$ where $Q=N \, q$ is the total charge and *L* is a characteristic length of the system (for instance, length of a square region) can be approximated as a discrete sum of terms, $I_N=\sum _{i<j}^{N} 1/r_{ij}/N^2$ where $r_{ij}$ is dimensionless given in units of *L* (thus, $r_{ij}$ is of order one) [For details, see Eqs. (22), (23) and pertaining discussions in Ref.^35^]. The contribution coming from the sum $\sum _{i<j}^{N} 1/r_{ij}$ is of the form $\alpha N^2$ where $\alpha$ is a finite number^36^. After dividing by $N^2$ we obtain the zero-th order finite approximation value to the (dimensionless) exact self-energy. In a nutshell, this leading term contribution in the thermodynamic limit is proportional to the total number of charged particles squared (to the power of two). However, based on our notation in Eq. (9), the dimensionless energy term $B^{min}_{N \times N}$ (given in the standard units of energy appropriate for a dipole–dipole interaction), has a leading term contribution in the thermodynamic limit that is proportional to the total number of dipoles (not number squared). Note the notation used in this section which is such that $N^2$ represents the total number of dipoles. This means that the dependence is very different (total number squared for Coulomb forces versus total number for dipolar forces) and, thus, one cannot say that the two systems mirror each other. One may add other terms as a first-order correction to the continuum limit approximation for the energy of a system of electric charges following various arguments^37^ or simply conjecturing them, but this is not the key point. The key point here is that the dependence of the electrostatic energy estimator as a function of the number of particles in an electrostatic system is not the same as its counterpart in a dipolar system. The correct dependence that we identified in Eq. (10) was obtained only after processing a large set of numerical data. As a result, it represents a novel postulated ansatz that is found to be excellent, only a-posteriori. On the other hand, the idea of the decomposition approach of the energy has its inspiration from the work in electrostatics although we had our doubts that it may work so well. Therefore, it is important to remark that the validity of the energy decomposition method for the dipole–dipole interactions is a novel unexpected result since there is no apriori reason even to believe why such a scheme should work for a dipolar interaction ($\propto 1/r^3$) that is highly anisotropic. It is enlightening to show in this work that this energy decomposition approach (with the appropriate quantitative tweaks already mentioned) works so excellently also for the dipole-dipole interaction self-energy as far as the procedure is concerned.

**Figure 3:**
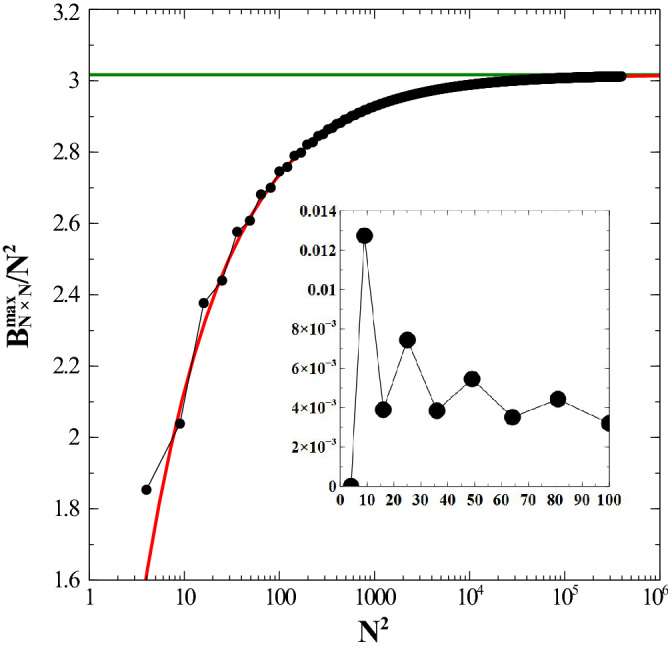
Dependence of the dimensionless energy (per dipole), $B^{\max }_{N \times N}/N^2$ (which represents an upper bound to the true maximum energy) as a function of the total number of dipoles, $N \times N$. The inset depicts the quantity $Q\,\equiv \,(U^{\max }_{N\times N} \,-\, B^{\max }_{N\times N}) / U^{\max }_{N\times N}$, which acts as a figure of merit for quantifying how good the bound $B^{\max }_{N\times N}$ is as a function of $N\times N$. The fit is represented by the solid line (red). Logarithmic scale used for the *x* axis. Energy is given in units of ${\frac{\mu _0}{4\pi } \frac{\mu ^2}{a^3}}$. See text for details. (Image created with Veusz 1.18; https://www.veusz.github.io).

Let us now follow with the calculation of the maximum possible energy. Starting from the finite cases, we noticed that the most important feature of the system is that the 2D system of dipoles will tend to resemble a specific ensemble of four dipole unit cells all pointing outwards with an orientation angle, $\theta$ such that $\tan \theta \,=\,\pm 1$. With other words, this arrangement is such that $\theta _{kl}\,=\,\pi +(-1)^l \frac{\pi }{4}$ for *k* even, and $\theta _{kl}\,=\,(-1)^{l+1} \frac{\pi }{4}$ for *k* odd. With this optimal configuration, the computation of $B^{\max }_{N\times N}$ is straightforward. The evolution of $B^{\max }_{N\times N}/N^2$ as a function of the number $N^2$ of dipoles is shown in Fig. 3. Notice that due to the shape of our plaquettes (2D square system) and the nature of the interaction in Eq. (2), the difference between each value and the asymptotic one is more pronounced for an even number $N^2$ of dipoles.

Also, in the corresponding inset of Fig. 3, one notes that the relative difference between the energy upper bound, $B^{max}_{N\times N}$ and the real one, $U^{max}_{N\times N}$ behaves in a somehow non-monotonic fashion but overall decreasing with increase of $N^2$. A similar analysis of the energy contributions as in the minimum energy case in Eq. (10), leads to the following results: $\{ {\mathcal {V}}\,=\, 3.01716 \,\pm \,6.291e-007;\, {\mathcal {S}}\,=\,-2.8072 \, \pm \,0.0003063\}$. Again, our numerical results show that the maximum energy per dipole in the thermodynamic limit, that is, ${\mathcal {V}}$, tends to the value 3.01716 *exactly*.

Once the range of possible energies is found, one may wonder about the particular form of the configuration for the state with minimum energy. In order to characterize it, we distort all the angles in the ground state by the same amount $\Delta \theta$ (in degrees), and numerically confirm that the ensuing dependence of the perturbed energy, $E(\Delta \theta )$ follows a *perfect* cosine law, given by $(-3.01716 - 2.54944)/2 \cos (2\pi \Delta \theta /180)\,+\,(3.01716 - 2.54944)/2$. What is surprising is that this perturbation allows the $E(\Delta \theta )$ to range from the minimum to the maximum possible energy in a continuous fashion. Incidentally, there are two angles (42.6 and 137.4) for which $E(\Delta \theta )$ is exactly zero.

**Figure 4:**
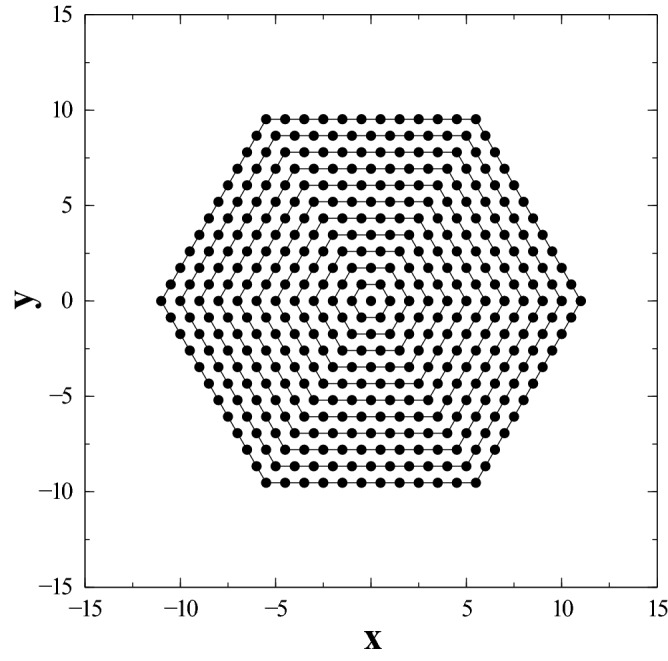
Shell structure of the growing sample of interacting dipoles in a planar hexagonal lattice. See text for details. (Image created with Veusz 1.18; https://www.veusz.github.io).

### Hexagonal lattice

**Figure 5:**
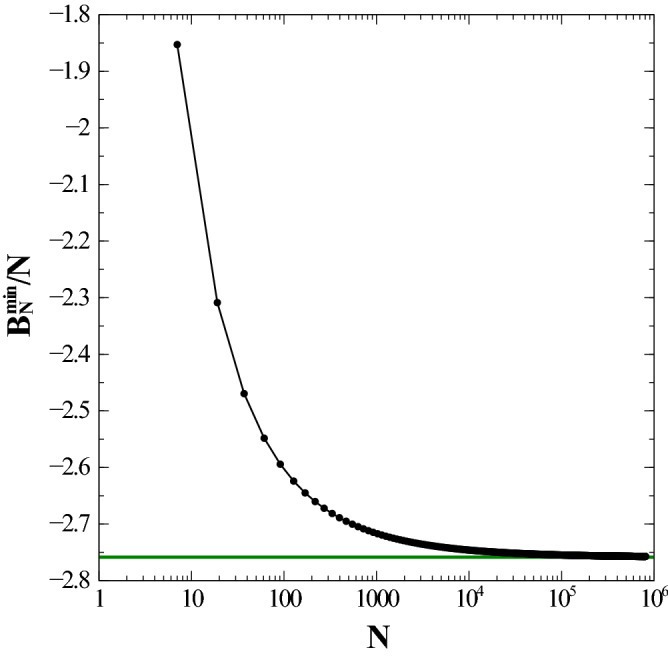
Dependence of the dimensionless energy per dipole, $B^{min}_{N}/N$ (which represents a lower bound to the true hexagonal ground state energy) as a function of the total number of dipoles, *N*. Logarithmic scale used for the *x* axis. Energy is given in units of ${\frac{\mu _0}{4\pi } \frac{\mu ^2}{a^3}}$. See text for details. (Image created with Veusz 1.18; https://www.veusz.github.io).

In order to study this configuration, we grow the system of interacting dipoles by adding closed layers of hexagonal shells. The concomitant structure is depicted in Fig. 4. Thus, in the same spirit as for the 2D simple square lattice, we grow the system of dipoles shell after shell. Instead of using brute force for each given *N* where *N* now represents the total number of dipoles, we employ a convenient numerical strategy that boosts the computation time for the total minimum or maximum energy calculations. Since we are adding compact layers, once we grow the plaquette from an initial hexagon to a brand new layer, the energy is computed according to the following steps: (i) energy among the interacting dipoles inside the new shell; (ii) energy between the dipoles of the new shell and the core ones; and (iii) energy in (i) and (ii) is added iteratively to the previously calculated one (the core). The computation done in this fashion for each shell optimizes the use of the computer time. Let us now consider the calculation of minimum energy. Unlike the 2D square lattice scenario, the hexagonal lattice case is of extraordinary complexity. No general rule could be at initially inferred from numerical optimization or symmetries. However, by comparing the results with optimal larger clusters, we obtained a compact expression which in turn suggests an extraordinary result involving a hidden six-fold symmetry of the system. By postulating an energy functional form of the type:

12 $$\begin{aligned} B^{\min }(x)\,\equiv \,{\mathcal {V}}\,x\,+\, {\mathcal {S}}\,\sqrt{x}\,+\,\alpha , \end{aligned}$$

we obtain the following values for the fitting constants: $\{ {\mathcal {V}}\,=\,-2.75854 \,\pm \,8.223e-009;\, {\mathcal {S}}\,=\,1.22402\, \pm \,7.662e-006; \, \alpha \,=\,3.30666 \, \pm \, 0.001497\}$. The results, depicted in Fig. 5 for $B^{min}_{N}/N$ as a function of *N* are in excellent agreement with Eq. (12), and provide for the first time, to the best of our knowledge, the ground state energy value of a 2D hexagonal lattice, $-2.75854$ with exact decimal digits for this level of accuracy.

**Figure 6:**
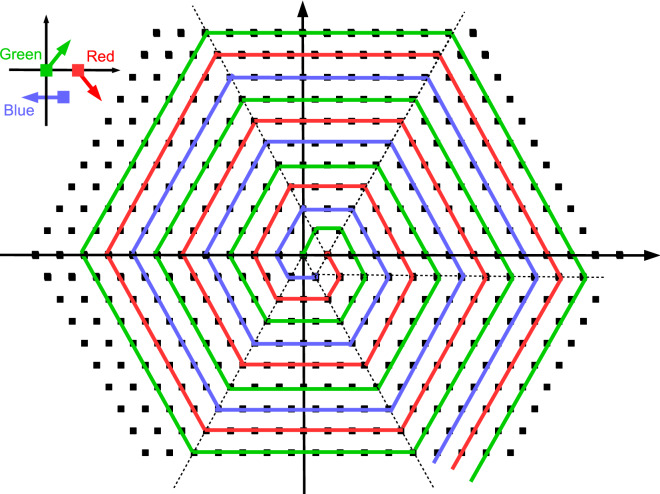
Ground state configuration for the interacting dipoles in the hexagonal case. See text for details. (Image created with OpenOffice 4.1.5; https://www.openoffice.org).

The ground state configuration is depicted in Fig. 6. The three embedded spirals conform an overall state with a nonzero total dipole moment, *M*. Since the total number of dipoles *N* goes as $3 \, n \, (n-1)+1$ for each new layer *n* added ($n=1$ correponds to a single dipole at the center, $n=2$ represents a shell of 6 dipoles, and so on) and the modulus of the total dipole moment *M* is easily found to be $(2n-1)\mu$ ; ($n\ge 2$), we have:

13 $$\begin{aligned} M \, = \, \frac{\mu }{3}\, \sqrt{12N - 3}. \end{aligned}$$

Thus, for a finite number of dipoles in a 2D hexagonal lattice, the ground state seems to possess a non-zero total magnetization *M*.

**Figure 7:**
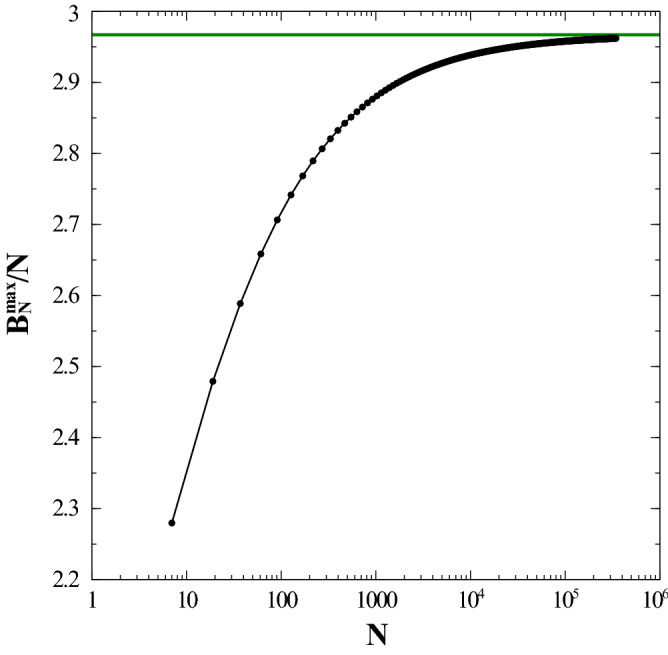
Dependence of the dimensionless energy per dipole, $B^{max}_{N}/N$ (which represents an upper bound to the maximum energy for the hexagonal lattice) as a function of the total number of dipoles, *N*. Logarithmic scale used for the *x* axis. Energy is given in units of ${\frac{\mu _0}{4\pi } \frac{\mu ^2}{a^3}}$. See text for details. (Image created with Veusz 1.18; https://www.veusz.github.io).

When considering the case of maximum energy, the ensuing equilibrium configuration is far simpler: for a fixed row *j*, $\theta = (-1)^j \,\frac{\pi }{2}$ (or its opposite) for all dipoles in the column *i*. With other words, the $x-y$ plane is divided in alternating stripes of dipoles, each stripe having all its dipoles pointing up or down. By postulating an energy functional of the type:

14 $$\begin{aligned} B^{\max }(x)\,\equiv \,{\mathcal {V}}\,x\,+\, {\mathcal {S}}\,\sqrt{x}\,+\,\alpha , \end{aligned}$$

we obtain the following results: $\{ {\mathcal {V}}\,=\,2.96697 \,\pm \,3.952e-008;\, {\mathcal {S}}\,=\, -2.88752\, \pm \,2.41e-005; \, \alpha \,=\,-0.566968 \, \pm \, 0.003083 \}$. The results shown in Fig. 7 for $B^{max}_{N}/N$ as a function of *N* are in excellent agreement with Eq. (14).

Admittedly, the particular form for the minimum energy state for the hexagonal lattice case is definitely puzzling. This state possesses a total magnetization per dipole that goes as $1/\sqrt{N}$, as opposed to the expected behavior 1/*N* in the thermodynamic limit. We cannot invoke any sort of topological transition for this scenario since such arguments are typically reserved to describe the ground state of quantum systems where degeneracy is a crucial element. This means that a distortion of the minimum energy state is mandatory here in order to shed some light on the system. The calculations for the 2D hexagonal lattice case return a perturbed energy, $E(\Delta \theta )$ of the form $(2.74388-2.75854)/2\,\cos (2\pi \Delta \theta /180) - (2.74388+2.75854)/2$. Again, we obtain a cosine dependence.

Differently from the 2D square lattice scenario, this perturbation does not explore the entire spectrum of energies, ranging only between − 2.75854 and − 2.74388. This fact is helpful to characterize the particular form of the minimum energy state in the hexagonal lattice case, but at the same time does not allow us to *truly* understand the particular topology of the concomitant state.

### Honeycomb lattice

**Figure 8:**
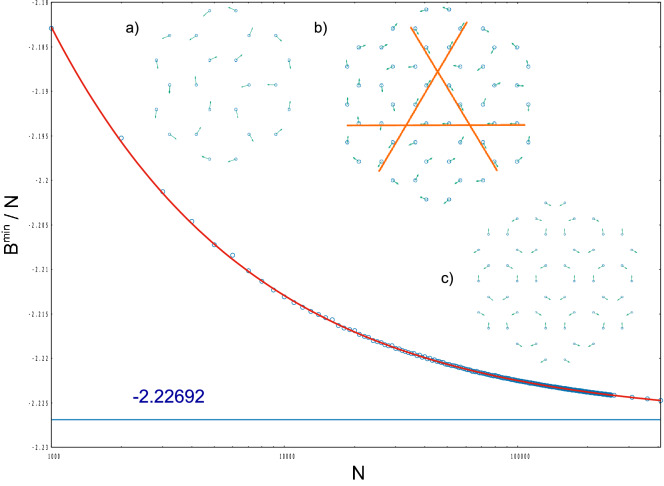
Plot of the evolution of the minimum energy per dipole in the hexagonal honeycomb lattice, $B^{min}/N$ as a function of the total number of dipoles, *N*. The solid curve represents the fit to the computed energies. The minimum energy per dipole in the thermodynamic limit is given by the horizontal line. (**a**) A cluster of $N=24$ dipoles with the minimum equlibrium energy. (**b**) The minimum energy configuration can now be detected for $N=54$ dipoles. (**c**) The maximum possible energy configuration counterpart. Energy is given in units of ${\frac{\mu _0}{4\pi } \frac{\mu ^2}{a^3}}$. See text for details. (Image created with OpenOffice 4.1.5; https://www.openoffice.org).

The honeycomb lattice is one of the twelve Archimedean tessellations or 1-uniform tilings that cover the plane using regular hexagons. The difference with the hexagonal tiling is that the vertex at the center is missing. In this case, and following our procedure, a sufficiently big sample of dipoles unveils the disposition of the dipoles for the minimum energy configuration after the optimization of the energy has occurred. Having said that, let us now illustrate in some detail the intricacies of the applied computational method. We start with a sample of $N=24$ dipoles as shown in Fig. 8a. One can appreciate that the finite-size effects dominate with $B^{min}/N=-2.00913252$ and the total dipole moment given by $2.30430229 \, \mu$. At this stage, no pattern can be grasped from the minimization procedure. However, one can now appreciate the ensuing equilibrium configuration of dipoles if the size of the system grows up to $N=54$ dipoles as depicted in Fig. 8b. Having identified the correct configuration, it is now possible to compute the minimum energies per particle which are depicted as empty circles in Fig. 8. The concomitant non-linear regression returns the minimum energy per dipole in the thermodynamic limit found to be $-2.22692 \,\pm \,3.249 \times 10^{-6}$, which is a little bit less bound that for the case of a hexagonal lattice. The same procedure is valid if one is interested to obtain the maximum energy. For instance the maximum energy configuration for $N=54$ dipoles is depicted in Fig. 8c. For such a case, $B^{max}/N=2.16684221$ and the total dipole moment is $0.0171540182 \, \mu$. One can proceed in a similar manner to resolve all different types of lattices either in 2D or 3D, namely, all those studied in the present work.

**Figure 9:**
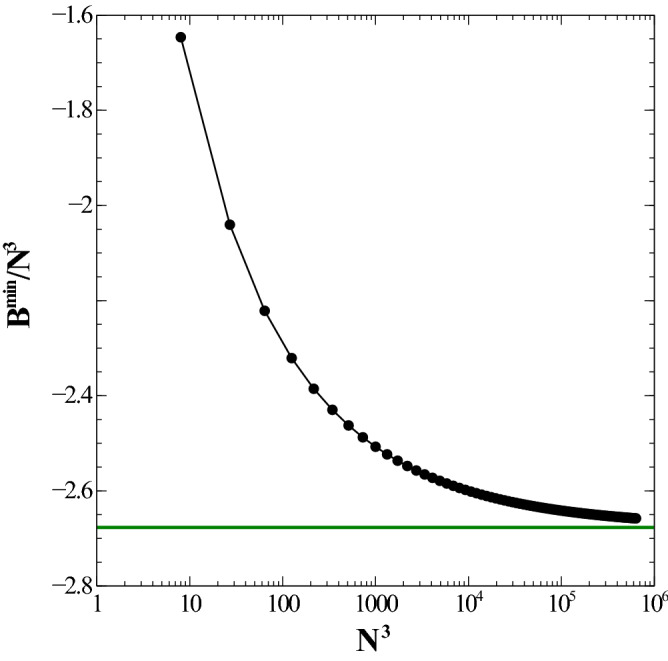
Dependence of the dimensionless energy (per dipole), $B^{\min }/N^3$ (which represents a lower bound to the ground state energy for the simple cubic lattice) as a function of the total number of dipoles, $N^3$. Logarithmic scale used for the *x* axis. Energy is given in units of ${\frac{\mu _0}{4\pi } \frac{\mu ^2}{a^3}}$. See text for details. (Image created with Veusz 1.18; https://www.veusz.github.io).

**Figure 10:**
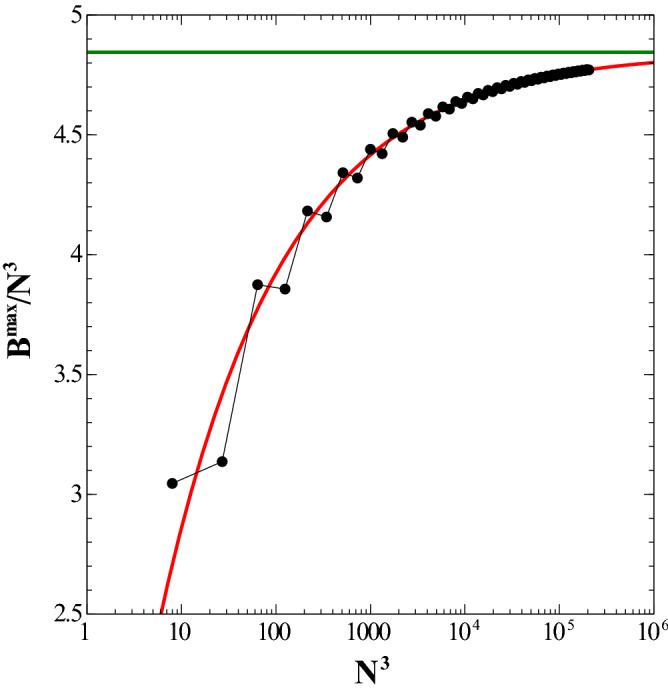
Dependence of the dimensionless energy (per dipole), $B^{\max }/N^3$ (which represents an upper bound to the maximum energy for the simple cubic lattice) as a function of the total number of dipoles, $N^3$. The fit is represented by the solid line (red). Logarithmic scale used for the *x* axis. Energy is given in units of ${\frac{\mu _0}{4\pi } \frac{\mu ^2}{a^3}}$. See text for details. (Image created with Veusz 1.18; https://www.veusz.github.io).

## Three-dimensional case

Let’s now consider a system of $N^3$ dipoles placed on the sites of a 3D simple cubic lattice with lattice spacing of *a*. This case together with that of face-centered and body-centered cubic lattices, was first considered by Luttinger and Tisza^30^. For this reason, this model serves as a good test to gauge again the accuracy and robustness of the numerical method that we employ. In the case of the ground state energy, a simple numerical exploration of the optimal minimum energy configurations leads to the following setup: position $x = i$, $y = j$, and *z* arbitrary (*i*, *j*, *k* positive integers including 0) and angles $\theta = [ (-1)^{i+j} - 1]\,\frac{\pi }{2}$ or its opposite value (global flip). As previously noted, all distances are dimensionless in units of cubic lattice length *a*. For this specific case, we considered the following guess for the functional form of the ground state energy:

15 $$\begin{aligned} B^{\min }(x)\,\equiv \,{\mathcal {V}}\,x\,+\, {\mathcal {S}}\,x^{2/3}\,+\,{\mathcal {L}}\,x^{1/3} \,+\, \alpha , \end{aligned}$$

where ${\mathcal {L}}$ is a new contribution arising from the cubical edge and *x* stands for the total number of dipoles in the finite cubic sample. After computing the results with the optimal configurations and adjusting the data to the form of Eq. (15) we obtain the following results: $\{ {\mathcal {V}}\,=\, -2.67679 \,\pm \, 1.665e-007;\, {\mathcal {S}}\,=\, 1.624 \, \pm \, 2.226e-005;\, {\mathcal {L}}\,=\, 0.6547 \, \pm \, 0.000851; \, \alpha \,=\, 0.560309 \, \pm \, 0.008808 \}$. The result for $B^{min}/N^3$ as a function of $N^3$ is shown in Fig. 9.

When it comes to the maximum energy, the ensuing configuration is a bit more involved. The optimal configuration is layered: in the first layer, the polar angle $\theta$ is $3\pi /4$ for all dipoles, whereas in the second one, $\theta$ is $\pi /4$ for all dipoles as well. This alternation is sustained all the way to the bulk. As far as the azimuthal angle $\phi$ is concerned, it is the same in all layers: all dipoles point outwards in the corresponding square subcell such that $\tan \phi =\pm 1$. More specifically, for $x/a\,=\,i$ even or zero, $\theta = \pi + (-1)^{j}\,\pi /4$, and $\theta = (-1)^{j+1}\,\pi /4 + 2\pi$, otherwise.

A similar calculation for the maximum energy $B^{\max }(x)$ returns the following values: $\{ {\mathcal {V}}\,=\, 4.84473 \,\pm \, 0.001591 ;\, {\mathcal {S}}\,=\, -4.27336 \, \pm \, 0.07999 \}$. The results for $B^{max}/N^3$ as a function of $N^3$ are depicted in Fig. 10. Differently from the minimum energy case, the maximum energy takes into account the parity of the number of dipoles, for instance, an odd number of particles in the edge makes the system “less repulsive”. Note that this energy difference between even and odd is blurred as we approach the bulk limit.

When comparing our results with the original ones in Ref.^30^, we not only do agree with them but we further increase the precision of the bulk energies. As a matter of fact, there are precise ways that we can speed-up our numerical computations such as to obtain results with any desired degree of accuracy.

**Figure 11:**
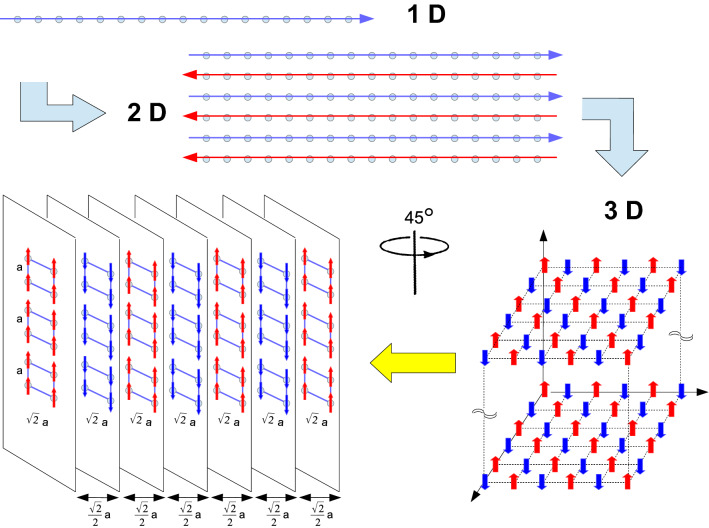
Evolution of the equilibrium configuration for 1D, 2D and 3D lattices. See text for details. (Image created with OpenOffice 4.1.5; https://www.openoffice.org).

## Conclusions

To conclude, we investigated the ground state energy of dipolar magnetic systems consisting of dipoles that are placed on the sites of some of the most common 1D, 2D and 3D crystal lattices. The 1D regular lattice case is well known and is shown only for completeness. For the case of 2D lattices, besides a simple square 2D lattice, we also considered much more complex 2D hexagonal and honeycomb lattices treating the dipoles as 2D XY spins that rotate only on the plane of the chosen 2D lattice. For the 3D case we considered a simple cubic lattice. The ground state of the model is degenerate by nature of the dipole–dipole interaction. For simulations with a finite number of dipoles, the ordering of various types of metastable domains depends on the boundary conditions and the size. As a result, this problem is a very challenging one to solve.

We used a new computational method to search for minimum and maximum energy values that represent, respectively, lower and upper bounds to the true energy per dipole corresponding to the system in the thermodynamic limit for all cases considered. While the 1D case is quite simple and does not deserve much attention, the 2D and 3D systems are not obvious. The calculations for a 2D square lattice and a 3D cubic lattice allow us to ascertain the validity and accuracy of our numerical method. This is the reason why we think it is appropriate to report them in this work. Having these results, allows us to have confidence on the approach and allows us to implement more detailed studies on rather complex hexagonal and honeycomb 2D lattices where new findings emerge.

In all cases, when exploring the ground state energies (minimum energy configurations) for common 2D and 3D lattices, we observe a regularity in the patterns on how the dipoles arrange themselves when they interact. This regularity is schematically shown in Fig. 11, where we see that the minimum energy configuration for simple lattices follows a clear pattern from 1D to the next one.

The minimum and maximum energy values are inferred from the finite clustered configurations ensuing step by step from numerical results. If not exact, they constitute excellent energy bounds to the values in the thermodynamic limit. Although we cannot provide a rigorous mathematical proof that the configurations are optimal as the number of dipoles increases indefinitely, we have obtained enough numerical evidence to assume that, indeed, they are. In fact, we found that the lower energy bounds that we obtained are very accurate for all the cases where analytical results are available, typically, 2D square and 3D cubic lattices, which serve to gauge the numerical accuracy of this novel computational approach.

At this juncture, it important to note that the philosophy behind the computations in our work unveils a brand new approach to obtain minimum/maximum equilibrium energies different from that of Luttinger–Tizsa method or Monte Carlo simulations. Our approach is rather simpler in the sense that we infer the minimum/maximum equilibrium energy configurations with a reasonable size of the sample of dipoles, which are taken in the most symmetric way according to the lattice symmetry. Once this configuration is known, the computation for larger and larger number of particles *N* is performed. Making use of the energy decomposition method, and regarding the unknown coefficients as free parameters, they can be adjusted to the computed energies for different *N* via a non-linear regression method. Finally, the leading term in the energy decomposition is obtained, that is, the energy per dipole, in $N\rightarrow \infty$ limit. This quantity is obtained with an extraordinary degree of accuracy as can be evidenced by looking at the convergence of the energy values, namely, the respective straight horizontal line for all figures in all dimensions.

The results that we obtained are in agreement with those in Ref.^30^ where the minimum energy was approximately calculated by using a method that reduced the large system to a small set of properly chosen class of cubic arrays for 3D simple cubic, body-centered cubic and face-centered cubic lattices. Our approach is different and, as a result, we are able to go beyond the cubic symmetry. Such is the case of 2D hexagonal lattices where we uncovered an interesting configuration for the minimum energy state represented by a set of three embedded spirals.

Generally speaking, it is expected that in typical situations the overall system shows super-paramagnetic behaviour in presence of dipole–dipole only interactions. The reason for the paramagnetic behavior is easily explained in the 2D simple square lattice by looking at the configuration of a special four-dipole structure consisting of adjacent dipoles at angles of the form $\pi \pm \pi /4$ and so on. However, the case of a 2D hexagonal lattice is much more exotic as illustrated by the appearance of a minimum energy state configuration with an unexpected evolution of the magnetization in the thermodynamic limit (its magnetization per dipole going as $1/\sqrt{N}$). In this sense, our method is able to uncover subtler and finer details that uniquely pertain to more complicated lattices^38,39^. This case also deserves further attention because of its relation to novel technological applications. In recent years, numerous improvements of surface technology in ultra thin films have unveiled various magnetic patterns with different symmetries as a function of temperature and lattice structure^40–42^. The magnetization distribution within such patterns describes topological defects such as vortices, domains, and walls^40–42^. Although real materials must be dealt quantum mechanically, such topological effects can also be explained in classic terms by evoking the long range dipole–dipole interaction. Topological quantum matter is another aspiring research field concerned with non-collinear spin textures in addition to spintronics. The most prominent example in this category is the magnetic skyrmion^43^, a whirl-like nano-object. Its topological protection gives it an enormous stability even at small sizes, which makes it a potential carrier of information in future data storage devices^44–46^. The existence of spin textures in quantum systems appeals to the exploration in full detail of the existence of their classical counterparts based solely on dipole–dipole interactions. It appears that these structures do not seem to occur in any other lattice but the 2D hexagonal/triangular one as we obtained in our case study. It also important to point out in this regard that, when our computational method is applied to these models, it leads to a better value for the hexagonal vortex configuration. Such a vortex state is expected to be very close to the minimum energy state, of the spiral configuration that we uncovered since this one has a considerable low energy of $-2.758545$. The vortex state is very robust and, as far as our numerical computations are concerned, does not depend on the boundary of the system, as opposed to spurious configurations that do not survive in the thermodynamic limit. Another remarkable property of the spiral state is that, among all subgroups of the symmetry group of the hexagonal lattice, only this one occurs in practice.

## Data Availability

The data are available upon request at jbv276@uib.es.
